# Saharan Dust Deposition May Affect Phytoplankton Growth in the Mediterranean Sea at Ecological Time Scales

**DOI:** 10.1371/journal.pone.0110762

**Published:** 2014-10-21

**Authors:** Rachele Gallisai, Francesc Peters, Gianluca Volpe, Sara Basart, José Maria Baldasano

**Affiliations:** 1 Departament de Biologia Marina i Oceanografia, Institut de Ciències del Mar, CSIC, Barcelona, Spain; 2 Istituto di Scienze dell'Atmosfera e del Clima, Roma, Italy; 3 Earth Sciences Department, Barcelona Supercomputing Center-Centro Nacional de Supercomputación, BSC-CNS, Barcelona, Spain; 4 Environmental Modelling Laboratory, Technical University of Catalonia, Barcelona, Spain; University of Yamanashi, Japan

## Abstract

The surface waters of the Mediterranean Sea are extremely poor in the nutrients necessary for plankton growth. At the same time, the Mediterranean Sea borders with the largest and most active desert areas in the world and the atmosphere over the basin is subject to frequent injections of mineral dust particles. We describe statistical correlations between dust deposition over the Mediterranean Sea and surface chlorophyll concentrations at ecological time scales. Aerosol deposition of Saharan origin may explain 1 to 10% (average 5%) of seasonally detrended chlorophyll variability in the low nutrient-low chlorophyll Mediterranean. Most of the statistically significant correlations are positive with main effects in spring over the Eastern and Central Mediterranean, conforming to a view of dust events fueling needed nutrients to the planktonic community. Some areas show negative effects of dust deposition on chlorophyll, coinciding with regions under a large influence of aerosols from European origin. The influence of dust deposition on chlorophyll dynamics may become larger in future scenarios of increased aridity and shallowing of the mixed layer.

## Introduction

Aerosols have major impacts on weather and climate regulations [1], [2] and even on crop production [3]. Atmospheric desert dust may travel large distances from its source and has been proposed to have ocean production regulation effects over geological times scales [4]. The Mediterranean Sea (hereafter Med) atmosphere is subject to the continuous injection of Saharan and Middle East mineral dust particles [5]. The deposition of these mineral particles supply numerous macro and micro- nutrients to the ocean surface [6]–[14] and some authors consider it as the major source of “new” nutrients [15] for system production.

Calculations show that the atmospheric input of nutrients in the Med is of the same magnitude as riverine inputs [16]–[18], thus playing a significant role in the regulation of the nutrient balance of the basin at decadal or longer time scales [19], [20]. The contribution of atmospheric deposition can be especially important and efficient in oligotrophic environments such as the Med, which has a marked stratification period and a pronounced nutrient limitation [21]. The deposition of some of these soluble compounds on surface waters may influence biological production, at least during certain events [9], [22], [23]. Dust deposition spreads over vast areas and dilutes into the water column often preventing the potential effects on system production to be unequivocally detected at ecological time scales. Experiments and observations in low nutrient – low chlorophyll areas have so far shown mixed results [24]–[26]. Reasons may include a tremendous variability in dust nutrient bioavailability content [27]–[29] and a relatively small increase of the background nutrient concentration when vertical mixing is active and represents the major source of nutrients [21] as well as a rapid transfer of increased primary production to other trophic levels and a variety of plankton community structures and physiological states.

Given the episodic nature of dust events, an additional complication may reside in the human capacity of detecting the dust event deposition with sufficient space-time resolution in order to build a statistically significant dust event database. Previous attempts used satellite-derived aerosol optical thickness (AOT) as a proxy of dust in the atmosphere to infer the deposition events [26], [30], [31]. Dust generally travels from several hundreds to thousands of meters high in the atmosphere, making this approach not quantitatively adequate for discerning between transport and deposition. Deposition is measured *in situ* at a few terrestrial (mainland and islands) sites, which are extremely valuable for ground truth validation but are dependent on local conditions, making generalizations hard to draw especially towards the open ocean. Here, we employ a state-of-art atmospheric transport and deposition model, the BSC-DREAM8b model [32], which has been validated [33]–[35] and gives the power of having aerosol deposition data over the whole Med basin with daily temporal resolution. A previous study showed the potential positive effects of dust deposition on SeaWiFS-derived chlorophyll (Chl) in the Med [32]. However, in the Med, the used NASA OC4v4 algorithm falls far short to retrieve Chl with accuracy smaller than 100%, casting doubts on the relationships found. Here we extend this approach by relating deposition to SeaWiFS Chl using the Med-specific algorithm MedOC4 [36]. When we think of dust deposition, we tend to think about very large events, those that are obvious in true color images or that we recognize because we find our cars covered with red dust, but the truth is that, to some extent, there is Saharan dust in the atmosphere over the Mediterranean almost continuously and deposition does not occur only during large events but also when atmospheric aerosol concentrations are not so high. Thus, rather than focusing on single events or experiments, we take a correlational approach using an 8-year data time series in order to find relationships between Chl dynamics and dust deposition over the Mediterranean Sea.

## Methods

### Chlorophyll data

SeaWiFS HRPT Level-1A data (2000–2007) were collected at the Istituto di Scienze dell’Atmosfera e del Clima of Rome, Italy, and processed up to Level-3 using the MedOC4 regional algorithm (http://www.myocean.eu/web/69-myocean-interactive-catalogue.php) [36]. This algorithm takes into account the peculiar blue-green ratio of Med waters. Level-3 Chl data, with a native 1 km resolution, were log_10_-transformed averaged, over a period of eight days, and regridded over the 1° resolution grid of the basin (179 cells, see Table S1). A previous study showed that it is recommended not to use 8-d averages when computing correlation analysis between Chl and dust events [26]. To account for the possible contamination by atmospheric dust mimicking chlorophyll, here, before averaging over the period of eight days, the quality of the entire Chl dataset was carefully checked by i) applying all the SeaDAS Level-2 processing masks and flags (http://oceancolor.gsfc.nasa.gov/VALIDATION/flags.html), ii) removing all isolated pixels, iii) removing all pixels exceeding 3 standard deviations within a moving box of 3×3 pixels, and iv) by applying a median filter over all remaining good pixels. This procedure increases the confidence level on data quality, with the only shortcoming of reducing the number of observations with respect to the NASA standard processing. The time series of daily observations was temporally binned into periods of 8 days. This results into 45 bins up to the 360^th^ day of the year. The last bin was computed with the remaining 5 days, and in the case of leap years, with the remaining 6 days. The climatic mean is then calculated across years for each of the natural 8-d time periods.

### Dust deposition

For the present study, a dust deposition simulation from the BSC-DREAM8b model (http://www.bsc.es/earth-sciences/mineral-dust/catalogo-datos-dust) model [33], [37] was used for the period between 1 January 2000 and 31 December 2007, over the Med basin. BSC-DREAM8b tracks mineral dust particles from their sources in the Sahara and Middle East regions. Output, after being log_10_-transformed, was provided for the same space and time resolution as for chlorophyll. A low cut-off threshold (10^−8^ Kg m^−2^ d^−1^) is applied to the numerical deposition output from BSC-DREAM8b since the dataset showed numerically correct but physically unrealistic low value spikes [32]. The model main features were described in detail in Pérez et al. [37] and Basart et al. [35]. It has been used for dust forecasting and as a dust research tool in North Africa and the Med [32], [38]–[40]. Several studies have checked its performance [33], [41], concerning both the horizontal and vertical extent of the dust plumes in the Med Basin. The model daily evaluation with near-real time observations is conducted at the Barcelona Supercomputing Center, and includes satellite data (MODIS and MSG) and AERONET sun photometers. BSC-DREAM8b has also been validated and tested over longer time periods in the European region [34], [42], [43] and against measurements at source regions [44].

### Aerosol Optical Thickness

AOT at 865 nm data were derived from SeaWiFS radiometer measurements and they were downloaded from the Giovanni database (http://gdata1.sci.gsfc.nasa.gov/daac-bin/G3/gui.cgi?instance_id=ocean_8day). We acquired 8-d averaged, 9 km resolution product from 2000 to 2007. Similarly to Chl and deposition data, AOT data were log_10_-transformed and regridded over the 1° resolution grid of the basin, with the same temporal binning. This was done for the same 179 1°×1° cells as for chlorophyll. It should be noted that AOT contains information of total aerosol particles in the atmosphere, not only of particles from Saharan origin. However, over much of the Mediterranean Sea most particles are indeed of Saharan origin [45].

### Statistical analyses

Pearson’s correlation coefficient (*r*) was calculated between chlorophyll concentration, modeled dust deposition and AOT time series for each grid cell. Significance was considered at p<0.05 using Student’s t-test. In addition, the degrees of freedom used for significance testing were adjusted to take into account the possible presence of autocorrelation in the time series. The number of effective independent observations, N*, were calculated as described in Pyper el al. [46]. Correlations were computed both for the entire series and for each season. The same analyses were performed after seasonally detrending the data by subtracting the climatic mean at each time series data point. The r^2^ of the correlation in a cell is the variance explained by the correlation in that cell. The minimum, maximum and average variance-explained values (expressed in %variability) were calculated for the population of cells with a p<0.05.

## Results and Discussion

**Figure 1:**
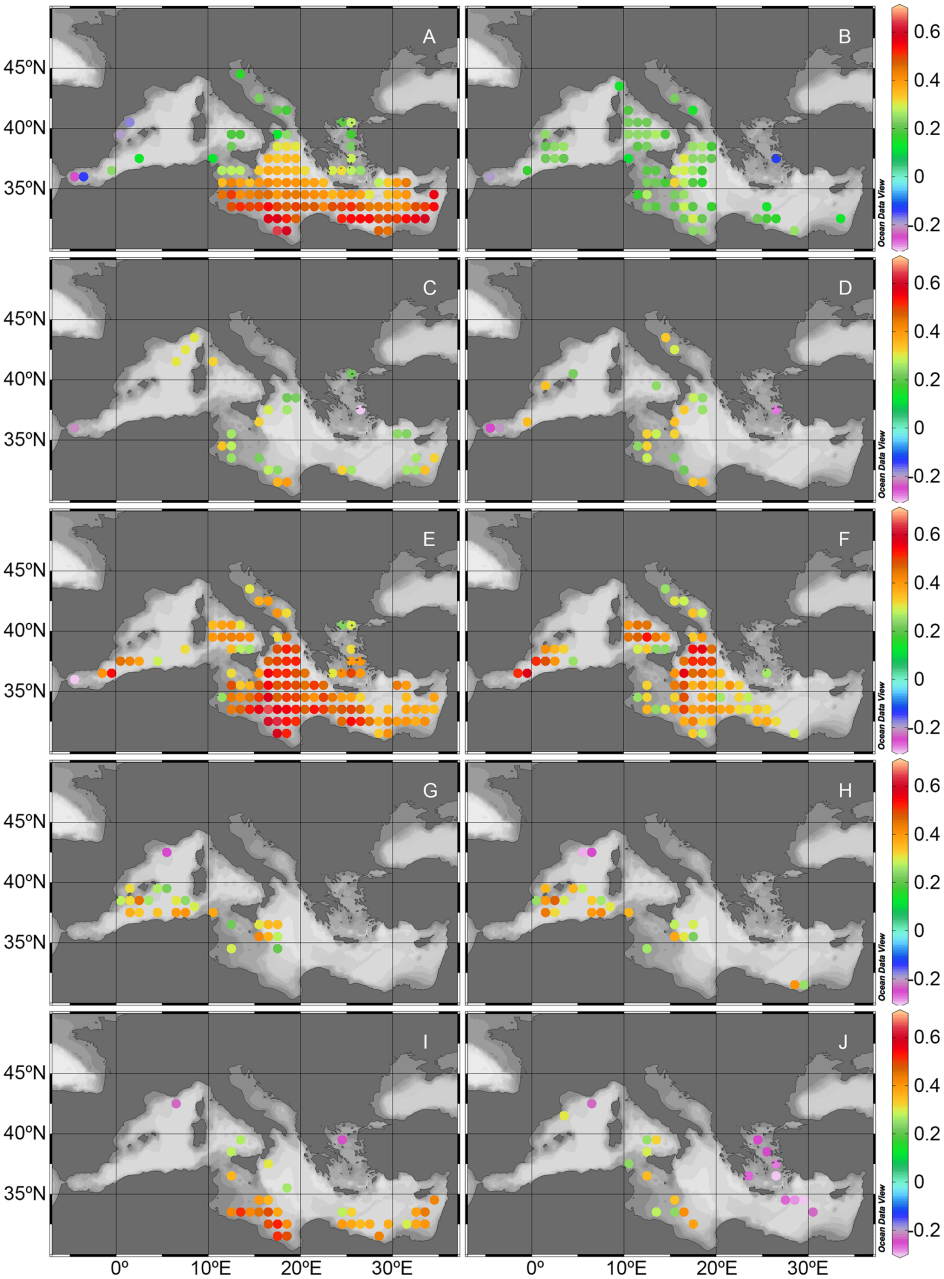
Correlation between chlorophyll concentration and dust deposition. Statistically significant (p<0.05) correlation coefficient (r) between chlorophyll concentration and dust deposition (left panels) and between the seasonally detrended chlorophyll concentration and the seasonally detrended dust deposition (right panels) for the whole time series and for different seasons. Panels: a, b) annual; c, d) winter (January to March); e, f) spring (April to June); g, h) summer (July to September) and i, j ) autumn (October to December).

We have found statistically significant positive correlations between surface chlorophyll and mineral dust deposition in large areas of the Med, covering 64% of the analyzed surface and located mainly in the Central and Eastern basins (Fig. 1) and with a clear south to north gradient in correlation intensity from 0.63 to 0.12. Significant negative correlations (r from −0.15 to −0.25) are observed in only 4 cells located in the Alboran Sea and in the eastern coast of Spain. Positive correlations can be found during all seasons, although it is in spring when we see the largest effects with correlations ranging from 0.22 to 0.65 mainly in the Central, Eastern and Southwestern Med. The Western and Central Med also show regions with positive correlations in summer, while in autumn there are some areas affected in the Central and Eastern Med. Most of the Med phytoplankton variability (>80%) is well explained by the variability of the mixed layer depth [47], and especially the winter-spring mixing bringing nutrient-rich deep waters to the surface. Thus, at least part of the explained variability between our deposition and chlorophyll time series must be due to the partial matching of the annual cycles of both variables.

**Figure 2:**
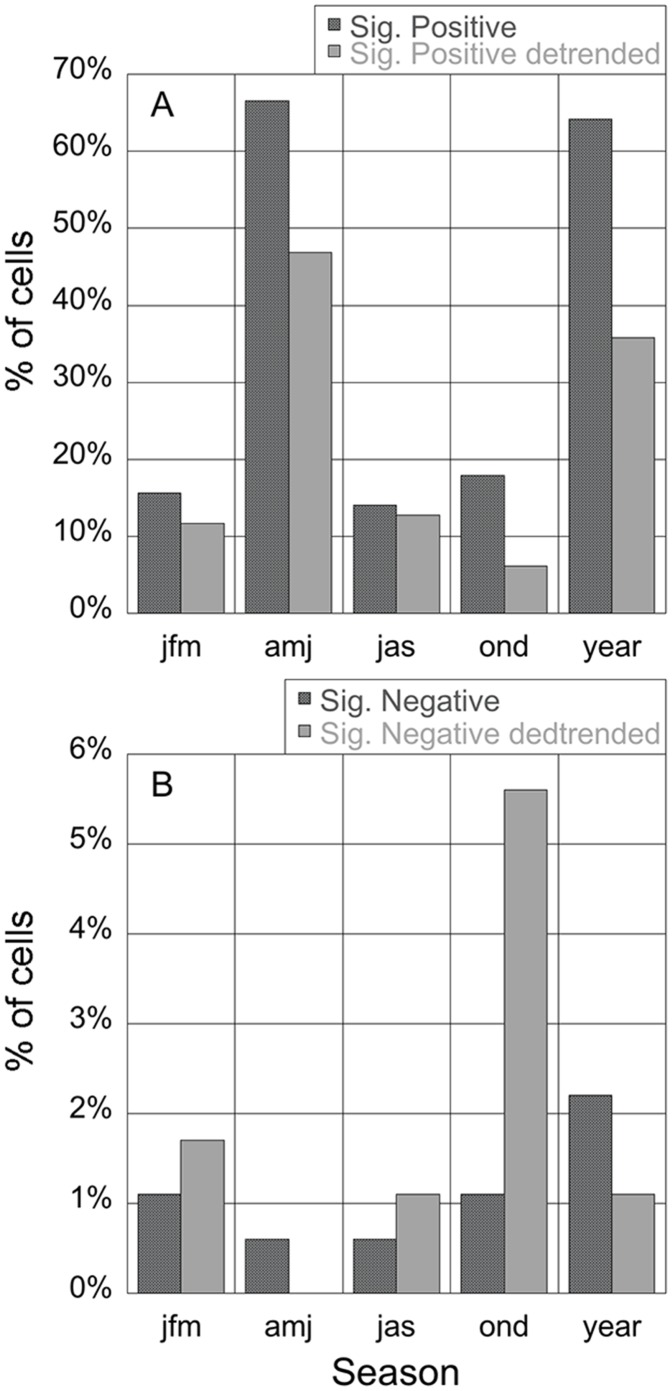
Percentage of cells showing significant correlations between chlorophyll and deposition. Left panel: positive correlations. Right panel: negative correlations. Red bars represent non seasonally detrended data and blue bars seasonally detrended data.

The relationship between the seasonally detrended data of chlorophyll and dust deposition, that represents more of a response of short-term chlorophyll peaks to dust outbreaks, is somewhat weaker in intensity and in area covered. Largest positive correlations are found in the Central Med (from 0.13 to 0.32) (Fig. 1). Again, it is in spring where the largest impacted area is found, mainly in the Central Med and extending into the Eastern Med and Southwestern Med with r ranging from 0.24 to 0.58. The Western Med shows the largest area affected in summer. This is not surprising given that the seasonal dust event frequency peaks during spring in the Central-Eastern Med, and during summer over the Central-Western basin [26]. Seasonally detrended data tend to slightly increase the number of cells showing significant negative correlations and decrease the number of significantly positive correlated cells (Fig. 2). It is in autumn when we see the largest number of negatively correlated cells (6% of analyzed surface) and located mainly in the Aegean Sea and extending southeasterly of Crete.

**Figure 3:**
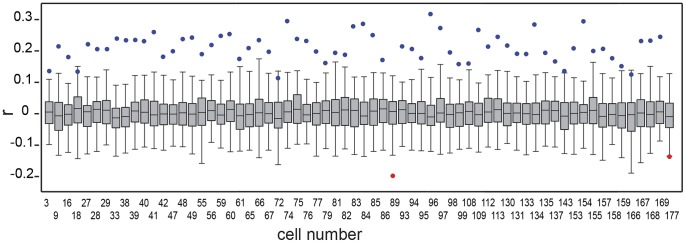
Analysis of the chance of significant correlations being spurious. Comparison between the box plots of the distribution of correlation coefficients between synthetic seasonally detrended chlorophyll time series and seasonally detrended dust deposition model outputs (N = 100) and the actual observed correlation between the seasonally detrended chlorophyll and the seasonally detrended dust deposition model outputs (dots). Data is shown only for those cells showing significant (p<0.05) observed correlations. Dots in blue represent significantly positive correlations and red significantly negative correlations. Box plots show the median, the grey box englobing all data between the 25 and 75 percentiles, and the range between the smallest and largest values that are not outliers. Starting from the detrended data of the cells that show statistically significant correlations between detrended chlorophyll and deposition (Fig. 1b), synthetic Chl time series with the same mean and standard deviation (normal distribution) as the original detrended chlorophyll time series, were computed for each cell. The correlation between these synthetic Chl time series and the modelled dust deposition were computed. For each cell, this process was repeated 100 times, and the probability distribution functions (PDFs) of the correlations were then obtained and presented as box plots.

For the seasonally detrended data, we checked that the correlation values were not caused by chance. We generated synthetic seasonally detrended chlorophyll time series with the observed mean and standard deviation for each cell. Correlations were computed with dust deposition model outputs, and the process repeated 100 times (Fig. 3). The observed significant correlations were compared to the distribution of the synthetic correlations for each cell, and in all cases they were statistically different with an α<0.001 and a power (1-β) undistinguishable from 1. This confirmed the non-spurious nature of the relationships between dust deposition and non-seasonal chlorophyll time series.

Bulk Saharan dust deposition over the Med is not straightforwardly related to dust travelling in the atmosphere (Fig. S1). Meteorological conditions and wind patterns at different times of the year often have large amounts of dust (AOT) travelling at altitude with little deposition [48]–[50]. AOT and dust deposition show positive correlation in the Western Med (Fig. S2.) especially in spring and summer with correlated areas shifting depending on the season. The Eastern sub-basin presents the highest correlations in spring (from 0.23 to 0.51) and the Central Med (Tyrrhenian Sea, Sicily channel and Dardanelle strait) in autumn. Once the data are seasonally detrended, deposition events are more related to AOT events, both when the whole series is considered and when the data are analyzed for the different times of the year (Fig. S2). With respect to non-detrended data, seasonally detrended data show main increases in correlation and correlated area in the Central Med for most of the year, as well as in the Eastern Med in autumn. Some overall hotspots appeared in the Alboran and in the Tyrrhenian Sea and around Crete, where the correlations ranged from 0.36 to 0.48.

The annual cycles of chlorophyll and AOT do not match (Fig. S1). The maximum chlorophyll concentrations occur in winter and minima coincide with the summer months. On the contrary, the highest AOT is found in summer and the minimum in autumn. Overall, AOT and chlorophyll (Fig. S3) show no significant correlations in the Med, except for some areas near the African coasts, where the correlation is negative (from −0.28 to −0.36). While no correlations are evident between AOT and chlorophyll there are significant correlations between seasonally detrended AOT and chlorophyll data (Fig. S3). A plume of higher correlation, with r-values between 0.33 and 0.39, appear in the northern part of Cyrenaica region with an extension up to the south of Italy. The best match between both series was found in summer (Fig. S3). Volpe et al. [36] ground truthed the chlorophyll satellite estimates with in situ measurements and concluded that the atmospheric correction was appropriate. In addition, we compared the data from the chlorophyll measurements at the DYFAMED station (1998–2007) with SeaWiFS estimates corrected with a regional algorithm giving a slope of ∼1 (logDYF = 0.0129+1.0497·logSW; Adjusted R^2^ = 0.68; N = 91; p<0.001). Moreover DYFAMED chlorophyll was unrelated to AOT, providing further evidence of the independence between satellite measurements of chlorophyll and AOT. Aerosols travelling over a certain area are not necessarily depositing. When a deposition event is occurring, it should coincide with high aerosol content in the air (AOT), thus if we find relationships between dust deposition events and non-seasonal chlorophyll peaks it is also logical to expect that chlorophyll is related to AOT, while the non-detrended AOT data show little or no relationship.

As mentioned before, the largest positive correlations between dust deposition and chlorophyll occur around the Central and Eastern Med. Calculations [8], [20], [51] and experiments [11], [20], [24] tell us that aerosol deposition effects on primary production should be small in most situations and thus we do not expect African dust deposition in general to explain a large portion of chlorophyll variability. Accordingly, positive significant correlations between mineral dust deposition of Saharan origin and chlorophyll do explain only a 1 to 10% (average 5%) of chlorophyll variability for seasonally detrended and a 1 to 40% (average 16%) for non-detrended data although it may be higher for certain seasons (Table 1). It should be noted that the explained variability does not provide direct information of the magnitude of chlorophyll impacted.

**Table 1:** Percentage of observed chlorophyll variability explained by modeled dust deposition.

	Non-detrended data				Seasonally detrended data			
Season	N	Min	Max	Average	n	Min	Max	Average
Annual	115	1.4	40.2	16.4	64	1.3	10.1	4.7
Winter	28	4.3	15.1	7.8	21	4.4	12.6	8.5
Spring	119	5.0	41.6	19.1	84	5.7	33.3	15.0
Summer	25	4.2	19.6	10.2	23	4.7	21.3	11.1
Autumn	32	6.5	26.9	15.6	11	5.4	16.5	9.6

Number of cells (n) with significantly (p<0.05) positive correlations. Minimum (min), maximum (max) and average percentage of chlorophyll variability explained in the significantly positive cells.

Winter shows overall the lowest significantly positive correlations, while spring presents the highest. This is to be expected since the entrance of new nutrients should be mostly due to seasonal winter overturning and mixing of nutrient-rich deep waters with upper ocean surface waters, through a number of physical processes that increase vertical diffusion at certain moments. But even at times when nutrient concentrations are expected to be relatively high in the water, low concentrations and strong imbalances between N and P are often observed [52], [53], opening windows of opportunity for the nutrients from atmospheric deposition to have an impact in the sustainment of phytoplankton production. We can only speculate on the positive cause-effect relationship between aerosol deposition and chlorophyll in the Med at certain times. Terrestrial inputs through major rivers occur mainly in the Western Med [54], and atmospheric inputs may dominate nutrient supply at certain times [10], [55]. Phosphorus (P) limitation alleviation has often been invoked [8], [56] as the surface waters of the Med are among the most P-limited in the world [57]. Although aerosols show a disproportionally large ratio of nitrogen to phosphorus [18], potentially only exacerbating P-limitation, they do carry an amount of P that could be used by phytoplankton and bacteria, especially in spring and summer when the concentrations of this element in surface waters of the open Med are at their lowest. Guieu et al. [10] calculated that, if P is considered the limiting element for phytoplankton growth, atmospheric deposition could account for chlorophyll increases of ca. 0.2 µg L^−1^ in the upper mixed layer for a single large deposition event or for the average total deposition during the summer-stratified period. The Central and Eastern Med do not show the typical spring phytoplankton bloom and have been defined as no blooming areas [58]. The ultra-oligotrophic conditions [59] found in these areas should make them most responsive to external nutrient supplies. As is the case for high nutrient – low chlorophyll areas, micronutrients such as iron from aerosols have also been proposed to stimulate Med phytoplankton production under certain situations [60], albeit addition experiments have not shown a direct increase in dissolved iron (Fe) [61]. Fe in the mixed layer of the Mediterranean is found at concentrations from 0.13 to 2.7 nM [62], [63]. It seems though that Fe is, relative to the needs of plankton, in excess with respect to P in the Mediterranean [64]. Nevertheless, in a system where all elements are relatively scarce, responses to the combination of elements arriving through aerosol deposition, may be very complex, with elements becoming successively limiting in a chained reaction. Ridame et al. [65] found stimulation of nitrogen (N) fixation in dust pulse experiments, in general related to a primary alleviation of P-limitation. In their Central Med experiment though, they found high N-fixation stimulation unrelated to P- or Fe-limitation, further showing the complexity of the processes involved and the potential spatial and temporal variability. An initial stimulation of heterotrophic bacteria [25], [66] should not be discarded since these organisms have a potential advantage at low nutrient concentrations owing to their high surface to volume ratio. Secondarily, released nutrients from recycling could then stimulate phytoplankton processes. Contrary to the Eastern Med showing the lowest nutrient concentrations in the Mediterranean [59], the Central Med was found somewhat more responsive to dust deposition in the present study. Pey et al. [5] mention the Central Med as a transitional area, receiving a higher frequency of dust outbreaks than similar latitudes in the Western and Eastern Med. Additionally, the dust source areas are not homogeneous. The Libyan Desert is the main source of dust for the Central Med while the Eastern Med receives dust from Libya and from the Middle East [67]. Thus, positive correlations between dust deposition and surface chlorophyll seem to arise from the combination of areas of low nutrient concentrations with the right nature, timing and frequency of dust outbreaks.

Negative relationships between dust deposition and chlorophyll have been related to metal (mainly Cu but also Al) inhibition of phytoplankton growth [13], [68]. The toxicity of Cu in reducing phytoplankton growth rate has been shown in laboratory experiments (see [68] and references therein). A recent correlation study between chlorophyll and metals from onshore-measured aerosols in the Northwest Med shows negative relationships in the area under northerly wind (Tramontane) conditions [13]. These winds favor the transport of anthropogenic aerosols from Europe to the Med. Although most Cu pulses are anthropogenically derived, pulses originating in Africa showed effects on chlorophyll undistinguishable from those originating locally [13]. A reduction in chlorophyll growth of up to 20% can be seen along the French and Spanish coasts. In addition, Jordi et al. [13] argue that since Cu toxicity seems to be taxon specific, the summer phytoplankton community with a predominance of nanoflagellates over the less sensitive diatoms, is more vulnerable to atmospheric deposition. This is an area where we also see some negative correlations between the modeled deposition and chlorophyll. We only track Saharan mineral dust, while some of the high load of metals may be more related to local anthropogenic sources. Most of the large deposition events in the Northwest Med come in the form of wet deposition [69]. In our model, the deposition field only originates from Saharan and Middle East dust transport and does not account for local anthropogenic aerosol sources, but rain washes out the entire atmospheric column aerosol loading, no matter the origin. Results from our correlation analysis agree with previous more detailed local studies [13]. We also see a negative relationship between deposition and chlorophyll, both seasonally detrended, mainly in autumn in the Aegean region (Fig. 1). This area is affected by long-range transport of air pollutants from Eastern Europe [70] but it is also heavily impacted by anthropogenic emissions generated in Athens and Istanbul [71], [72]]. A high-density population together with a massive number of vehicles, many of them still using non-catalytic or old technology diesel engines, contributed to exceed the EU annual aerosol limit. The amount of Cu in these aerosols is high with an annual mean concentration between 0.013 and 0.22 µg m^−3^ ([73] and references therein). An estimated dry deposition flux of Cu over the sea ranges then between 22 and 380 µg Cu m^−2^ d^−1^ surpassing the threshold limit for Cu to inhibit phytoplankton growth rate according to [13] and [68].

## Conclusion

Desert dust storm events seem to be increasing in frequency and intensity [14], [69], [72]–[76] in the last decades, due to human activities and climate forcing. This means that the presence of aerosols over the Med is likely to increase with future aridity. Thus, it is important to understand basin level patterns in the response of Med biogeochemistry to aerosol deposition. Only a few studies [26], [30] have tried to analyze the potential links between aerosols in the air column and chlorophyll for the entire Med basin, with non definitive results. In this study, we use a modeled actual aerosol deposition product and show both positive and negative significant correlations with chlorophyll dynamics in certain areas and times of the year. Mineral dust from North Africa and the Middle East correlates to chlorophyll in large areas of the Med Sea. This is especially true for the Central and Eastern Med sub-basins, where Saharan dust deposition dynamics matches that of chlorophyll, particularly during spring. Here the atmospheric input may be an intrinsic part of the annual ecosystem dynamics. In terms of large dust outbreaks, chlorophyll best relates to aerosol deposition in the Central Med, extending both into the Eastern and Southwestern Med (Fig. S3). Some areas of the Western Med and Aegean Sea show negative correlations between chlorophyll and deposition in accordance with some recent findings of toxicity brought by metals in aerosols. As expected, dust deposition does not explain an overall large amount of chlorophyll variability since the main ecosystem production driver in the Med is the vertical mixing of nutrients from deep waters. Variability related to carbon to chlorophyll ratios, the consumption of biomass with a varying degree of coupling and the variable settling of primary production, are all additional sources of surface chlorophyll variability that we could not account for in our correlations and thus add to the noise. No matter how small significant correlations are, they are not distributed randomly in space and coincide with independent estimates that follow the same trend. Thus, albeit the mechanisms that affect chlorophyll through aerosol deposition cannot be pinpointed and may be indirect, non-unique, and dependent on local spatio-temporal conditions, our study shows a clear potential for effects at ecological scales. These effects should become more important in a future scenario with increased aerosols over the Med and a shallower mixed layer depth, owing to increased temperatures, over which aerosols may leach out nutrients.

## Supporting Information

Figure S1 **Seasonal average values of chlorophyll concentration, dust deposition and aerosol optical thickness.** Average chlorophyll concentration (left panels). Average dust deposition (central panels) and average aerosol optical thickness (right panels) for different seasons. Winter (a, b, c), spring (d, e, f), summer (g, h, i) and autumn (j, k, l).
(TIF)

Figure S2 **Correlation between dust deposition and aerosol optical thickness.** Statistically significant (p<0.05) correlation coefficient (r) between dust deposition and aerosol optical thickness (left panels) and between seasonally detrended dust deposition and seasonally detrended aerosol optical thickness (right panels) for the whole time series and for different seasons. Panels: a, b) annual; c, d) winter (January to March); e, f) spring (April to June); g, h) summer (July to September) and i, j ) autumn (October to December).
(TIF)

Figure S3 **Correlation between chlorophyll concentration and aerosol optical thickness.** Same as Fig. S2 but for chlorophyll concentration versus aerosol optical thickness.
(TIF)

Table S1 **Geographical coordinates for the 1°×1° grid cells analyzed in this study.** Coordinates refer to the central point of the cell. Latitudes are all North. Positive longitudes are East and negative longitudes West.
(DOCX)
