# Supplementary figures and images for: Saharan Dust Deposition May Affect Phytoplankton Growth in the Mediterranean Sea at Ecological Time Scales
Source: PLoS One. 2014 Oct 21;9(10):e110762. doi: 10.1371/journal.pone.0110762 (PMC4205005; doi:10.1371/journal.pone.0110762)

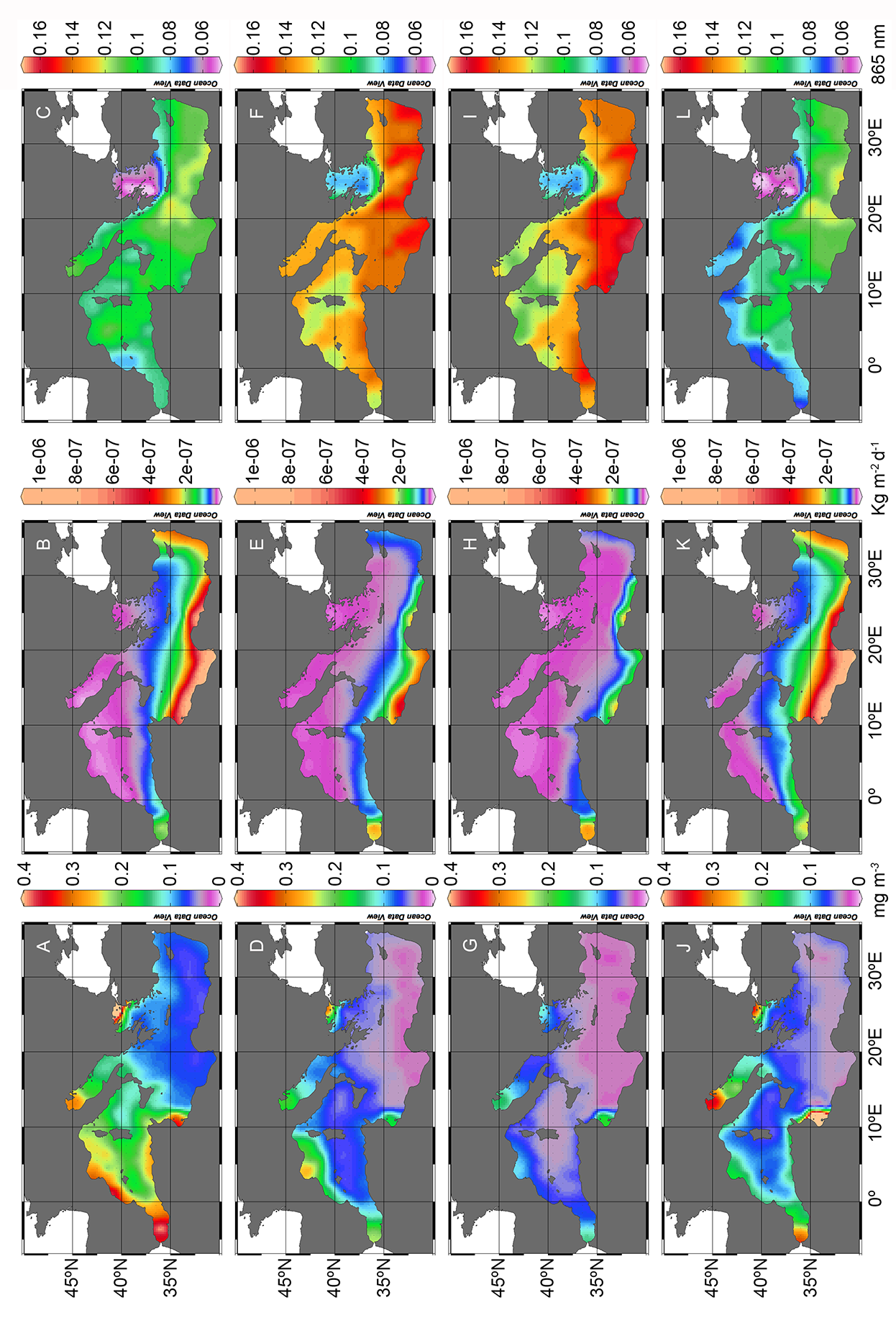

Supplement: Figure S1 — Seasonal average values of chlorophyll concentration, dust deposition and aerosol optical thickness. Average chlorophyll concentration (left panels). Average dust deposition (central panels) and average aerosol optical thickness (right panels) for different seasons. Winter (a, b, c), spring (d, e, f), summer (g, h, i) and autumn (j, k, l). (TIF) [file pone.0110762.s001.tif]

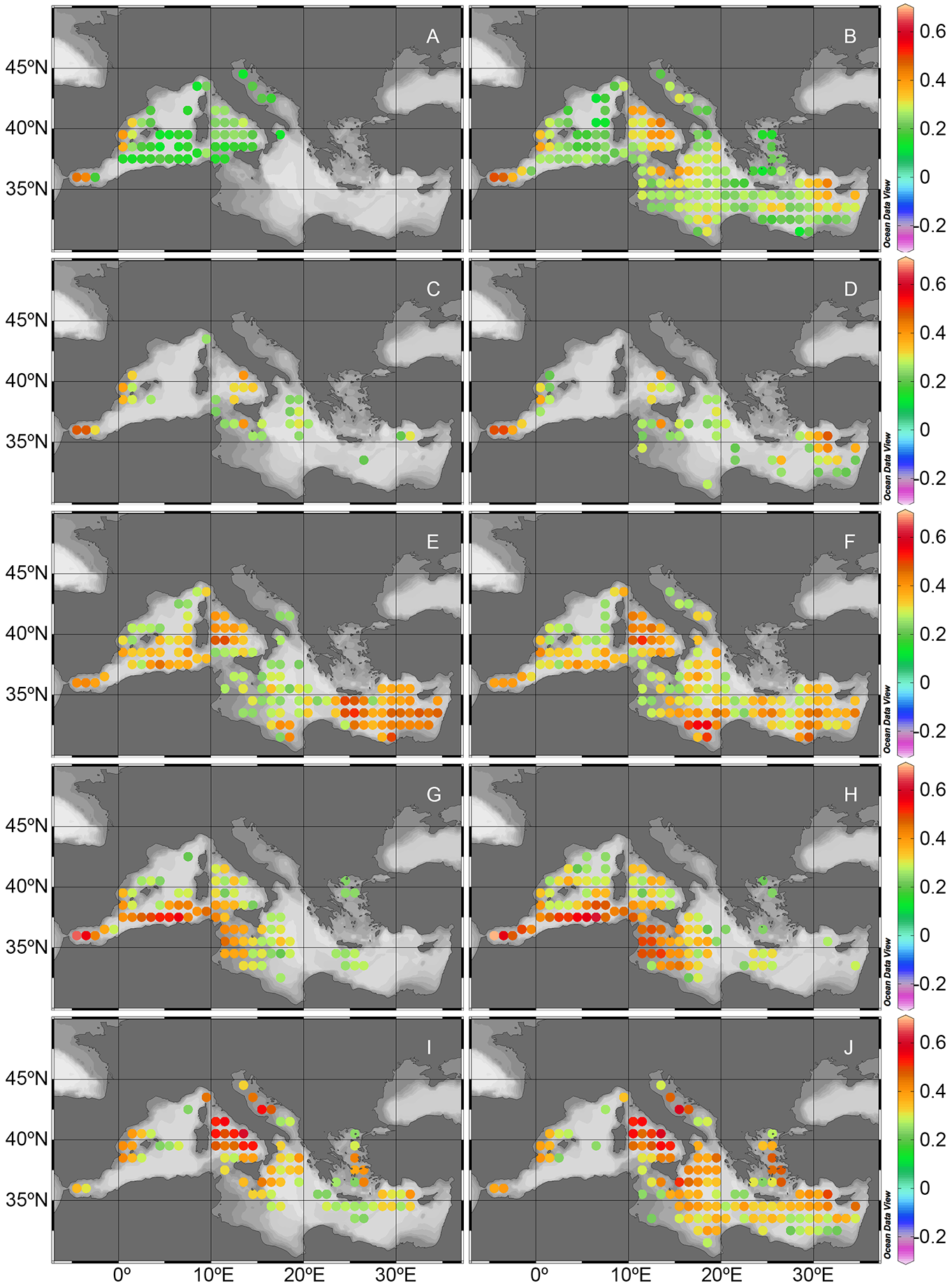

Supplement: Figure S2 — Correlation between dust deposition and aerosol optical thickness. Statistically significant (p<0.05) correlation coefficient (r) between dust deposition and aerosol optical thickness (left panels) and between seasonally detrended dust deposition and seasonally detrended aerosol optical thickness (right panels) for the whole time series and for different seasons. Panels: a, b) annual; c, d) winter (January to March); e, f) spring (April to June); g, h) summer (July to September) and i, j ) autumn (October to December). (TIF) [file pone.0110762.s002.tif]

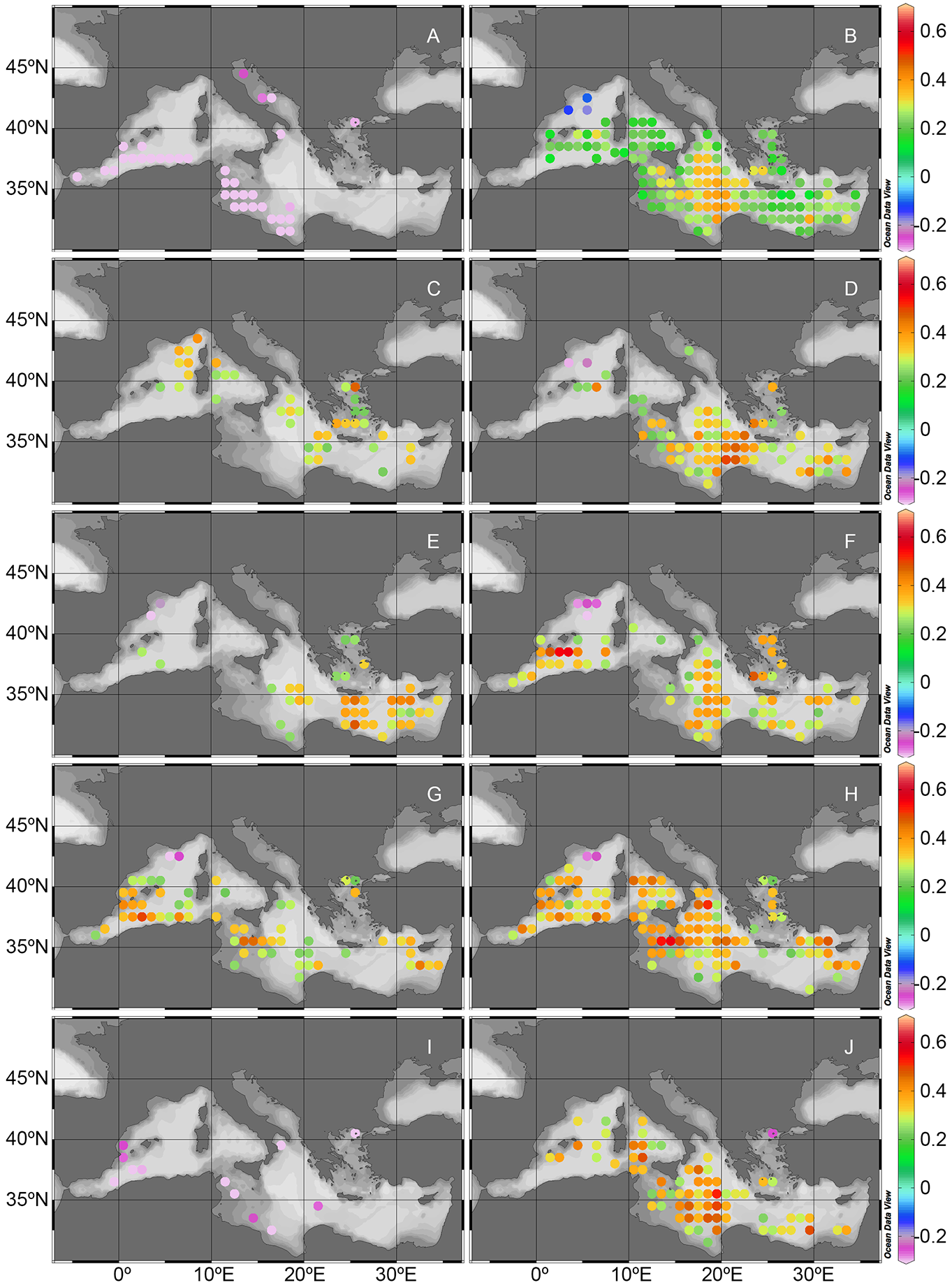

Supplement: Figure S3 — Correlation between chlorophyll concentration and aerosol optical thickness. Same as Fig. S2 but for chlorophyll concentration versus aerosol optical thickness. (TIF) [file pone.0110762.s003.tif]
