# Supplementary material for: Saharan Dust Deposition May Affect Phytoplankton Growth in the Mediterranean Sea at Ecological Time Scales
Source: PLoS One. 2014 Oct 21;9(10):e110762. doi: 10.1371/journal.pone.0110762 (PMC4205005; doi:10.1371/journal.pone.0110762)
Supplement: Table S1 — Geographical coordinates for the 1°×1° grid cells analyzed in this study. Coordinates refer to the central point of the cell. Latitudes are all North. Positive longitudes are East and negative longitudes West. (DOCX) [file pone.0110762.s004.docx]

| **Cell nº** | **Latitude** | **Longitude** | **Cell nº** | **Latitude** | **Longitude** | **Cell nº** | **Latitude** | **Longitude** |
| --- | --- | --- | --- | --- | --- | --- | --- | --- |
| **1** | 44.5 | 13.5 | **61** | 38.5 | 17.5 | **121** | 34.5 | 15.5 |
| **2** | 43.5 | 8.5 | **62** | 38.5 | 18.5 | **122** | 34.5 | 16.5 |
| **3** | 43.5 | 9.5 | **63** | 38.5 | 19.5 | **123** | 34.5 | 17.5 |
| **4** | 43.5 | 14.5 | **64** | 38.5 | 25.5 | **124** | 34.5 | 18.5 |
| **5** | 42.5 | 4.5 | **65** | 38 | 8.5 | **125** | 34.5 | 19.5 |
| **6** | 42.5 | 5.5 | **66** | 38 | 9.5 | **126** | 34.5 | 20.5 |
| **7** | 42.5 | 6.5 | **67** | 37.5 | 0.5 | **127** | 34.5 | 21.5 |
| **8** | 42.5 | 7.5 | **68** | 37.5 | 1.5 | **128** | 34.5 | 22.5 |
| **9** | 42.5 | 15.5 | **69** | 37.5 | 2.5 | **129** | 34.5 | 23.5 |
| **10** | 42.5 | 16.5 | **70** | 37.5 | 3.5 | **130** | 34.5 | 24.5 |
| **11** | 41.5 | 3.5 | **71** | 37.5 | 4.5 | **131** | 34.5 | 25.5 |
| **12** | 41.5 | 4.5 | **72** | 37.5 | 5.5 | **132** | 34.5 | 26.5 |
| **13** | 41.5 | 5.5 | **73** | 37.5 | 6.5 | **133** | 34.5 | 27.5 |
| **14** | 41.5 | 6.5 | **74** | 37.5 | 7.5 | **134** | 34.5 | 28.5 |
| **15** | 41.5 | 7.5 | **75** | 37.5 | 10.5 | **135** | 34.5 | 29.5 |
| **16** | 41.5 | 10.5 | **76** | 37.5 | 11.5 | **136** | 34.5 | 30.5 |
| **17** | 41.5 | 11.5 | **77** | 37.5 | 16.5 | **137** | 34.5 | 31.5 |
| **18** | 41.5 | 17.5 | **78** | 37.5 | 17.5 | **138** | 34.5 | 34.5 |
| **19** | 41.5 | 18.5 | **79** | 37.5 | 18.5 | **139** | 33.5 | 12.5 |
| **20** | 40.5 | 1.5 | **80** | 37.5 | 19.5 | **140** | 33.5 | 13.5 |
| **21** | 40.5 | 2.5 | **81** | 37.5 | 25.5 | **141** | 33.5 | 14.5 |
| **22** | 40.5 | 3.5 | **82** | 37.5 | 26.5 | **142** | 33.5 | 15.5 |
| **23** | 40.5 | 4.5 | **83** | 36.5 | -1.5 | **143** | 33.5 | 16.5 |
| **24** | 40.5 | 5.5 | **84** | 36.5 | -0.5 | **144** | 33.5 | 17.5 |
| **25** | 40.5 | 6.5 | **85** | 36.5 | 11.5 | **145** | 33.5 | 18.5 |
| **26** | 40.5 | 7.5 | **86** | 36.5 | 12.5 | **146** | 33.5 | 19.5 |
| **27** | 40.5 | 10.5 | **87** | 36.5 | 13.5 | **147** | 33.5 | 20.5 |
| **28** | 40.5 | 11.5 | **88** | 36.5 | 15.5 | **148** | 33.5 | 21.5 |
| **29** | 40.5 | 12.5 | **89** | 36.5 | 16.5 | **149** | 33.5 | 22.5 |
| **30** | 40.5 | 13.5 | **90** | 36.5 | 17.5 | **150** | 33.5 | 23.5 |
| **31** | 40.5 | 24.5 | **91** | 36.5 | 18.5 | **151** | 33.5 | 24.5 |
| **32** | 40.5 | 25.5 | **92** | 36.5 | 19.5 | **152** | 33.5 | 25.5 |
| **33** | 39.5 | 0.5 | **93** | 36.5 | 20.5 | **153** | 33.5 | 26.5 |
| **34** | 39.5 | 1.5 | **94** | 36.5 | 23.5 | **154** | 33.5 | 27.5 |
| **35** | 39.5 | 4.5 | **95** | 36.5 | 24.5 | **155** | 33.5 | 28.5 |
| **36** | 39.5 | 5.5 | **96** | 36.5 | 25.5 | **156** | 33.5 | 29.5 |
| **37** | 39.5 | 6.5 | **97** | 36.5 | 26.5 | **157** | 33.5 | 30.5 |
| **38** | 39.5 | 7.5 | **98** | 36 | -4.5 | **158** | 33.5 | 31.5 |
| **39** | 39.5 | 10.5 | **99** | 36 | -3.5 | **159** | 33.5 | 32.5 |
| **40** | 39.5 | 11.5 | **100** | 36 | -2.5 | **160** | 33.5 | 33.5 |
| **41** | 39.5 | 12.5 | **101** | 35.5 | 11.5 | **161** | 33.5 | 34.5 |
| **42** | 39.5 | 13.5 | **102** | 35.5 | 12.5 | **162** | 32.5 | 16.5 |
| **43** | 39.5 | 14.5 | **103** | 35.5 | 13.5 | **163** | 32.5 | 17.5 |
| **44** | 39.5 | 17.5 | **104** | 35.5 | 14.5 | **164** | 32.5 | 18.5 |
| **45** | 39.5 | 18.5 | **105** | 35.5 | 15.5 | **165** | 32.5 | 19.5 |
| **46** | 39.5 | 24.5 | **106** | 35.5 | 16.5 | **166** | 32.5 | 24.5 |
| **47** | 39.5 | 25.5 | **107** | 35.5 | 17.5 | **167** | 32.5 | 25.5 |
| **48** | 38.5 | 0.5 | **108** | 35.5 | 18.5 | **168** | 32.5 | 26.5 |
| **49** | 38.5 | 1.5 | **109** | 35.5 | 19.5 | **169** | 32.5 | 27.5 |
| **50** | 38.5 | 2.5 | **110** | 35.5 | 20.5 | **170** | 32.5 | 28.5 |
| **51** | 38.5 | 3.5 | **111** | 35.5 | 21.5 | **171** | 32.5 | 29.5 |
| **52** | 38.5 | 4.5 | **112** | 35.5 | 22.5 | **172** | 32.5 | 30.5 |
| **53** | 38.5 | 5.5 | **113** | 35.5 | 28.5 | **173** | 32.5 | 31.5 |
| **54** | 38.5 | 6.5 | **114** | 35.5 | 29.5 | **174** | 32.5 | 32.5 |
| **55** | 38.5 | 7.5 | **115** | 35.5 | 30.5 | **175** | 32.5 | 33.5 |
| **56** | 38.5 | 10.5 | **116** | 35.5 | 31.5 | **176** | 31.5 | 17.5 |
| **57** | 38.5 | 11.5 | **117** | 34.5 | 11.5 | **177** | 31.5 | 18.5 |
| **58** | 38.5 | 12.5 | **118** | 34.5 | 12.5 | **178** | 31.5 | 28.5 |
| **59** | 38.5 | 13.5 | **119** | 34.5 | 13.5 | **179** | 31.5 | 29.5 |
| **60** | 38.5 | 14.5 | **120** | 34.5 | 14.5 |  |  |  |
